# CTCF and cellular heterogeneity

**DOI:** 10.1186/s13578-019-0347-2

**Published:** 2019-10-11

**Authors:** Gang Ren, Keji Zhao

**Affiliations:** Laboratory of Epigenome Biology, Systems Biology Center, National Heart, Lung and Blood Institute, National Institutes of Health, Bethesda, MD 20892 USA

## Abstract

Cellular heterogeneity, which was initially defined for tumor cells, is a fundamental property of all cellular systems, ranging from genetic diversity to cell-to-cell variation driven by stochastic molecular interactions involved all cellular processes. Different cells display substantial variation in gene expression and in response to environmental signaling even in an apparently homogeneous population of cells. Recent studies started to reveal the underlying mechanisms for cellular heterogeneity, particularly related to the states of chromatin. Accumulating evidence suggests that CTCF, an important factor regulating chromatin organization, plays a key role in the control of gene expression variation by stabilizing enhancer–promoter interaction.

## Background

Heterogeneity was initially defined for “tumor heterogeneity” by Heppner 35 years ago [1], referring the observation that different tumor cells can show distinct morphological and phenotypic profiles [2]. The tumor heterogeneity was observed occurring at two different levels: inter-tumor heterogeneity and intra-tumor heterogeneity. It is believed that the intra-tumor heterogeneity could introduce significant challenges in human treatment strategies [3, 4]. Actually, heterogeneity is a widely spread phenomenon in all life systems, from genetic diversity to cell-to-cell variation in all cellular processes. In particular, cell to cell heterogeneity (or sometimes called variation) in gene expression has been described and investigated from bacteria to humans, which may be a key link between upstream genetic diversity and downstream phenotypic heterogeneity. Recent studies have indicated that cells even from an apparently homogeneous population show variation in expression and in response to environmental stimulations [5–7]. Transcription, a major step of gene expression control, is regulated by multiple factors in eukaryotic systems, including sequences of promoters and enhancers, nucleosome occupancy and position, epigenetic modification and long-range chromatin interaction [8–20]. Thus, the variation in gene expression in eukaryotic cells may result from numerous mechanisms including fluctuations of upstream regulators, such as promoter, enhancer, and insulator, temporal variations of epigenetic modification states or long-range interactions [14] or stochastic bursts of transcription [21]. Recent studies with new single-cell epigenomics techniques have revealed new insights into the underlying mechanism of cellular heterogeneity [16, 22–24]. In particular, enhancer–promoter interactions mediated by CCCTC-Binding Factor (CTCF) plays a critical role in the control of cell-to-cell variation in gene expression [14].

### CTCF acts as chromatin barrier and enhancer blocker

CTCF gene encodes a transcriptional regulator protein with 11 highly conserved zinc finger (ZF) domains which exhibit almost identical amino acid sequences among vertebrates, and more divergent in N and C terminals [25]. It is required for normal embryonic development and cellular differentiation [26–28]. The using of different combination of eleven ZF domains allows this protein to bind different DNA sequence and/or interact with various protein factors. Depending on the context, it can function as a transcriptional activator or repressor [25]. In early studies, CTCF was considered as a transcriptional repressor using reporter gene assays for the regulatory regions of chicken and human *c*-*Myc* genes [29, 30]. Soon after, it was found that it could act as a transcriptional activator at the Amyloid β-Protein Precursor TSS [31]. Later on, CTCF was found to have the enhancer blocking and/or barrier insulation activity at the chicken β-globin locus and at the imprinted control region (ICR) of the mammalian *H19/Igf2* locus [32–34]. This ability was defined by the capacity to block the communication between promoter and regulatory elements such as enhancers, and also the spread of repressive heterochromatin from adjacent genetic regions [35]. Consistent with this function, CTCF binding enriched at the boundary regions demarcates active and repressive chromatin domains marked by H2AK5Ac and H3K27me3 in human cells [36].

### CTCF contributes to higher-order genome organization

The mammalian genomes are organized into megabase-sized local chromatin interaction domains, the topologically associating domains (TADs) defined from Hi-C interactions [9]. TADs tend to tally with epigenetic domains, contain co-regulated genes, and are highly conserved across cell types and species [9, 15, 37, 38]. TADs can be divided into smaller domains with enhanced contact frequency, named DNA loops or sub-TADs, which are more variable across different cell types [11, 15, 39]. TAD boundaries are often associated with CTCF binding to its motifs of convergent orientation [11]. Disruption of the CTCF binding sites at TAD boundaries causes the loss of TAD structure and dysregulation of transcription of genes within the TADs [40–43], suggesting a critical role of CTCF protein in maintaining the TADs structure in genome. CTCF and Cohesin-mediated loop formation results in insulated chromatin domains, which is critical for the proper expression or repression of local genes involved in pluripotency or lineage specification in mouse ES cells [44]. Deletion of the CTCF target sites leads to inappropriate interaction of enhancers inside the neighborhood with genes outside the neighborhood and thus improper expression of relevant genes [44]. Similarly, the CTCF binding sites within the *Hox* gene clusters function to insulate adjacent chromatin domains during embryonic stem cell differentiation into cervical motor neurons. Deletion of CTCF binding sites results in the expansion of active chromatin into the repressive domain, causing *Hox* genes’ dysregulation [45]. A loop exclusion model was proposed to explain the requirement of two convergent CTCF motifs for loop formation [46–48]. In this model, two convergently bound CTCFs act as extrusion barriers, Cohesin complex serves as extruding factors. When Cohesin is halted in both directions by bound CTCFs, the loop is formed [46, 47]. Supporting this model, it was found that single nucleotide mutation of CTCF motif sequence or inversion of core motif DNA sequence of CTCF resulted in disruption of the TADs structure and dysregulation of nearby genes [49, 50]. The deletion or inversion of DNA sequence that disrupts a CTCF-associated boundary domain causes limb enhancer misplaced relative to TAD boundaries and drives ectopic limb expression in human limb malformations [20].

The contribution of CTCF to TADs structure was also shown by different strategies to control CTCF expression. Knocking down of CTCF expression using siRNAs not only reduced the intradomain interactions but also increased interactions between neighboring domains [51]. More recently, by acute and reversible depletion of CTCF using the auxin-inducible degron (AID) system in mESCs, Nora and colleagues elegantly demonstrated that CTCF is indispensable and dose-dependently required for looping between CTCF target sites and insulation of TADs. Depletion of CTCF eliminates CTCF-mediated DNA looping and TADs genome-wide [42].

### CTCF facilitates enhancer–promoter interaction

Although CTCF binding is enriched in TAD boundaries and important for TAD structure, CTCF binding sites are widespread in the genome and actually the vast majority of them are located within TADs [10, 12, 14, 35, 51–53] structure. Furthermore, these intra-domain CTCF binding sites are in the vicinity of potential enhancers of transcription, marked by P300 and H3K4me1, and thus may influence the activity of enhancers [14]. A chromosome conformation capture carbon copy (5C) study in human GM12878, K562 and HeLa-S3 cells found that a fraction of CTCF enriched distal elements significantly interact with gene promoters, which suggests that one of the main roles of CTCF in genome function may be to facilitate the interaction between regulatory sequences and promoters [54]. Since distal enhancers must physically contact with their target promoters to carry out their activity, the nearby CTCF molecules may bring enhancers to the vicinity of their target promoters [14]. CTCF can mediate the enhancer–promoter contact through the interaction between CTCF bound nearby enhancers and Cohesin loaded nearby promoters [46, 55, 56]. Liu et al. reported that regulatory elements-bound CTCF/cohesin can recruit the core promoter factor TAF3 and mediate its contact with promoters through TAF3-dependent loop formation in ES cells and depletion of CTCF reduces the efficient recruitment of TAF3 to distal regulatory elements, compromises endoderm differentiation marker gene expression, such as Gata4, Afp, and Apoa1 [57]. CTCF interacts with the enzyme poly-ADP-ribose (PARP1) itself to help establish inter-chromosomal contacts between active circadian loci and repressive chromatin at the lamina, thereby mediates circadian transcriptional plasticity. Furthermore, knockdown of CTCF expression counteracts both recruitment to the repressive lamina at envelope and circadian transcription [58]. Recently, we systematically profiled CTCF-mediated promoter-enhancer interaction in mouse primary Th2 cells by integrating CTCF ChIP-Seq and 3e Hi-C interaction data. We observed a positive correlation between CTCF binding and enhancer activities as indicated by H3K27ac, suggesting that CTCF binding influences enhancer activity. Furthermore, we found that active promoters exhibit significantly higher interaction with CTCF sites than silent promoters, and the enhancers that interacted with CTCF sites also exhibited significantly higher interaction with promoters, which confirmed that CTCF binding sites interact with their neighboring enhancers and facilitate the functional interaction between enhancers and promoters. Using shRNA knockdown of CTCF, we confirmed that CTCF contributes to the expression of lineage-specific genes by mediating the interaction between their enhancers and promoters. We further found that CRISPR/CAS9-mediated deletion of intra-domain CTCF binding sites significantly compromised the interactions between CTCF binding sites, promoters, and enhancers at *Thy1, Cd5* and *Runx3* gene loci, which, however, did not disrupt the TAD structure. Together, these results indicate that one major role of intra-domain CTCF binding is to mediate the interaction between enhancers and their target promoters [14].

### CTCF contributes to the control of cellular heterogeneity in gene expression

Although there is increasingly convincing evidence showing that CTCF critically contributes to the interaction between enhancers and promoters, depletion of CTCF protein in cells by either shRNA or AID leads to only modest expression changes of relatively small number genes at cell population level. Since the RNA-Seq and Western blotting assays measure the average gene expression level of a population of cells, the observed modest changes in gene expression may reflect one of two ways of gene expression change: (1) modest but similar changes in every cell and (2) little change in the majority of cells but substantial change in a fraction of cells. While the former is consistent with the homogeneous property of all cells, the latter informs the heterogenous property of a cell population. Using quantitative single-cell assays including fluorescence-activated cell sorting (FACS) and single-cell RNA-FISH, we monitored gene expression in each single cell and found that CTCF-bound T cell-specific genes GATA3, CD90, CD28, CD5 displayed significantly increased expression variation in CTCF depleted cells [14]. These results supported the cellular heterogeneous property of the cells and suggested an important role of CTCF in the control of gene expression heterogeneity. However, the increased cell-to-cell variation of expression by knocking down of CTCF could also be accounted for by the heterogeneous CTCF knockdown efficiency across different cells. Conclusive evidence came from the deletion of a specific CTCF binding site at *Thy1* locus, nearby a distal enhancer, using CRISPR/CAS9, which resulted in a significantly higher cell-to-cell variation of gene expression in the CRISPR knockout cells [14].

### CTCF contributes to cellular heterogeneity control by stabilizing enhancer–promoter interactions

Recent studies have demonstrated that sequences of promoters, nucleosome occupancy, epigenetic modifications and three dimensional genome organization all contribute to the regulation of gene expression in eukaryotic systems [8–18]. Consequently, gene expression variation may result from any fluctuation of above-mentioned factors, especially for CTCF mediated promoter-enhancer interaction. Recent studies indicated that intra-domain CTCF binding sites are frequently found in enhancer regions [12, 14, 51]. CTCF binds and brings distal enhancers, via interaction with Cohesin, to the vicinity of their target promoters [14]. The increased heterogeneity in gene expression by deletion of CTCF binding site at *Thy1* locus is correlated with decreased Thy1 promoter-enhancer interaction but not changes in the TAD structure, strongly suggest a model that CTCF binding near the enhancer region stabilizes the interaction between the *Thy1* promoter and its enhancers and thus reduces the cell-to-cell variation of *Thy1* expression. More studies to visualize the enhancer–promoter interaction in single-cells would be needed in future to prove this model. Next, we discuss other potential mechanisms that CTCF use to contribute to cellular heterogeneity.

### The methylation status of CTCF binding motif could affect cellular heterogeneity

DNA methylation can block CTCF binding in genome [34, 59–61]. The H19 imprinted control region (ICR) is an enhancer-blocking element required for imprinting of the *H19* and *Igf2* genes [62]. The conserved CTCF sites in HS1 and HS2 of the ICR are essential for the enhancer-blocking activity. The methylation of CTCF binding motifs of these sites abolishes CTCF binding and results in the loss of the epigenetic regulation of *Igf2* [59]. These observations are consistent with the constitutively methylated status on both alleles in Wilms tumors with loss of *Igf2* imprinting in humans [63]. Recently, Comparison of genome-wide occupancy patterns of CTCF with bisulfite sequencing data in 19 diverse human cell types, including normal primary cells and immortal lines reveal that 41% of variable CTCF binding is linked to differential DNA methylation, which is enriched at CTCF recognition sequence. Disruption of CTCF binding in immortal cell lines is associated with increased methylation at promoter sites [60]. Furthermore, the binding of CTCF is sufficient to effect a local demethylation state [64]. These data suggest that CTCF could contribute to expression variation via regulating dynamics of DNA methylation at regulatory regions. However, CTCF is not the originator of the unmethylated state at *Igf2/H19* gene locus [65], and also it is unclear whether demethylation facilitates subsequent CTCF binding or whether bound CTCF maintains an unmethylated domain. To test this, Liu and collages employed dCas9-Dnmt3a to target de novo methylation of CTCF motifs in mES cells. Targeting of dCas9-Dnmt3a to the CTCF binding site bordering the *miR290*, *Pou5f1* gene loops blocked CTCF anchoring, resulted in significantly increased interaction frequency between super-enhancers and newly activated genes (*Nlrp12, H2Q10*) in the neighboring loop, and accompanied by increased expression of Nlrp12, H2Q10 [61]. In humans, IDH mutations, which mis-regulates genome methylation and compromise CTCF binding, promote gliomagenesis by disrupting chromosomal topology and allowing aberrant regulatory interactions that induce oncogene PDGFRA expression [66]. These data demonstrate that the de novo change of the methylation state of specific CTCF anchor sites could interfere its insulator/looping function, which may result in increased noise of transcription.

### The mutation of CTCF binding motifs may increase expression heterogeneity

The mammalian cells have about 50,000 CTCF binding sites, with 10 to 20% located in TAD boundaries and 60 to 70% located in intra-domain regions [11, 12, 36, 44, 52, 67]. GWAS studies have identified numerous mutations in CTCF binding sites [68, 69] and these mutations could affect gene regulation by TAD organization or enhancer–promoter interactions mediated by CTCF and thus increase the variability of gene expression. However, this notion needs more supporting evidence from single cell studies.

### CTCF may contribute to cellular heterogeneity by effects on nucleosome positioning

Nucleosome positioning is an important chromatin feature that regulates gene expression [70–72]. The accessibility of critical regulatory regions in chromatin to transcription factors can be heavily hindered by the nucleosome structure and thus remodeling or removal of the nucleosome structure is required for gene activation [73, 74]. Recently, we analyzed genome-wide nucleosome positioning in hundreds of single mammalian cells and found that the cell-to-cell variation in nucleosome position is positively correlated with that in DNase hypersensitivity and transcription of underlying genes [16]. This study suggests that any factor that influences nucleosome positioning may contribute to the cellular heterogeneity in gene expression. Analysis of data from this study indicated that mutations of the CTCF motifs in the genome could result in decreased CTCF binding and nucleosome repositioning [16], which is consistent with the previous observation that CTCF could induces stable positioned arrays of nucleosome around its binding sites, and also significantly affects local chromatin accessibility during ES differentiation [75, 76]. Therefore, further investigation is needed to uncover the function of CTCF binding in nucleosome position variation in genome, which clearly, could also leads to expression heterogeneity.

### CTCF may contribute to cellular heterogeneity by regulating transcriptional pausing and alternative mRNA splicing

Alternative mRNA splicing is another source of cellular heterogeneity in mammalian cells. It is estimated that about 90% of human genes undergo alternative splicing of pre-mRNA [77]. The rate of RNA polymerase II transcription elongation influences splice site selection by the spliceosome, regardless the availability of splicing factors that detect cis regulatory elements [78]. It was reported that methylation of DNA sequence in the middle of a gene causes a decrease in Polymerase II elongation [79]. Further studies indicated that increased DNA methylation in exons is associated with increased splicing retention of alternative exons via MeCP2 pathways [80]. Other studies found that polymerase II tends to stall at CTCF/Cohesin binding sites in living human cells [81], which may increase the efficiency of pre-RNA splicing. Later on, it was found that the genome-wide CTCF binding at promoter-proximal regions well correlated with high polymerase II pausing indexes, and therefore, the effect of CTCF on RNA Pol II elongation may be widespread [82]. For example, In the mouse *Myb* locus, CTCF interferes with RNAPII elongation at its first intron, leading to low expression of the Myb [83]. Since CTCF binding affected by DNA methylation, the methylation status of CTCF binding motifs could regulate pre-RNA splicing. Indeed, Shukla et al. found that CTCF binds to exon 5 of *CD45* gene, pauses polymerase II elongation, results in the inclusion of exon 5 in mRNA; and DNA methylation inhibits CTCF binding to the target site near exon 5, consequently causes the exclusion of exon 5 in mature transcripts [84]. Therefore, it is highly likely that CTCF may also contribute to cellular heterogeneity by regulating transcriptional pausing and alternative mRNA splicing in mammalian cells.

### Cell cycle related dynamics of CTCF and CTCF DNA binding may contribute to cellular heterogeneity

Progression of cell cycle is associated with specific expression of a group of genes at distinct phases of the cell cycle. For example, *Cdh1* is expressed in G1 phase; histone genes are expressed in S phase; expression of the majority of genes is shut off in M phase. Thus, different phases of cell cycle create a kind of cellular heterogeneity within a population of cells. While it is not clear what is the role of CTCF in controlling the expression of the cell cycle specific genes, CTCF may be involved in the formation of globally distinct chromatin structure during cell cycle progression. It is well established that there are dramatical changes of chromosome organization in mitotic phase [85–87]. Interestingly, TADs and A/B compartments are lost during prometaphase [37, 88]. However, whether the loss of the high order genome structure is due to the loss of CTCF binding at the prometaphase stage is unknown. Recent data indicated that cell cycle dependent dynamics of CTCF DNA binding results in dynamics of factor binding and nucleosome positioning [89, 90]. Based on live cell imaging and genomics techniques, it was found that the dynamic changes of chromatin organization between interphase and mitotic phase, especially prometaphase, can be explained by loss and gain of genome wide CTCF binding, accompanied by the rearrangement of the nucleosomes flanking CTCF motifs [89]. The molecular mechanisms underlying this phenomenon may be related with the cell cycle associated CTCF protein level and phosphorylation status in cells [91–94]. Phosphorylation of CTCF greatly reduces its DNA binding capability, which could explain the observation that CTCF dissociates from chromatin during mitosis [92, 93, 95]. Taken together, these data suggest that cell cycle related CTCF abundance and its DNA binding dynamics may contribute to cellular heterogeneity during cell cycle progression.

### Perspectives

The emerging theme from recent studies is that cellular heterogeneity could be the output of nucleotide mutation, abnormal of histone modification, transcription factor binding, and also higher order chromosomal structures [2, 13, 41, 53, 83, 96]. CTCF is a well-studied chromatin protein, which may contribute to transcription regulation by a variety of different mechanisms including facilitating enhancer–promoter interaction, maintaining TAD structure, and influencing transcriptional elongation and splicing of pre-RNAs. Thus, any factor that modulates the CTCF activity in these processes may contribute to cellular heterogeneity. These include post-translational modification of CTCF, point-mutations of CTCF protein itself, CTCF-interacting proteins, mutation and methylation status of CTCF target motifs. In future, new/improved tools, particularly used for single-cell analysis of genome organization, regulatory factors binding, transcription state, and also epigenome information, are required to investigate the contribution of CTCF to cellular heterogeneity and its relevance to normal development and human diseases.

## Data Availability

Not applicable.

## References

[1] Heppner GH (1984). Tumor heterogeneity. Can Res.

[2] Marusyk A, Polyak K (1805). Tumor heterogeneity: causes and consequences. Biochimica Et Biophysica Acta-Rev Cancer.

[3] Reiter JG (2018). Minimal functional driver gene heterogeneity among untreated metastases. Science.

[4] Sottoriva A (2013). Intratumor heterogeneity in human glioblastoma reflects cancer evolutionary dynamics. Proc Natl Acad Sci USA.

[5] Moffitt JR (2018). Molecular, spatial, and functional single-cell profiling of the hypothalamic preoptic region. Science.

[6] Turajlic S, Sottoriva A, Graham T, Swanton C (2019). Resolving genetic heterogeneity in cancer. Nat Rev Genet.

[7] Yan T (2019). Multi-region sequencing unveils novel actionable targets and spatial heterogeneity in esophageal squamous cell carcinoma. Nat Commun.

[8] Giannini P, Braunschweig M (2009). DNA methylation patterns at the IGF2-H19 locus in sperm of Swiss Landrace and Swiss Large White boars. J Anim Breed Genet.

[9] Dixon J (2012). Topological domains in mammalian genomes identified by analysis of chromatin interactions. Nature.

[10] Phillips-Cremins JE (2014). Unraveling architecture of the pluripotent genome. Curr Opin Cell Biol.

[11] Rao SSP (2014). A 3D map of the human genome at kilobase resolution reveals principles of chromatin looping. Cell.

[12] Tang ZH (2015). CTCF-mediated human 3D genome architecture reveals chromatin topology for transcription. Cell.

[13] Bradner JE, Hnisz D, Young RA (2017). Transcriptional addiction in cancer. Cell.

[14] Ren G (2017). CTCF-mediated enhancer–promoter interaction is a critical regulator of cell-to-cell variation of gene expression. Mol Cell.

[15] Yu M, Ren B (2017). The three-dimensional organization of mammalian genomes. Annu Rev Cell Dev Biol.

[16] Lai BB (2018). Principles of nucleosome organization revealed by single-cell micrococcal nuclease sequencing. Nature.

[17] Platt JL, Kent NA, Kimmel AR, Harwood AJ (2017). Regulation of nucleosome positioning by a CHD Type III chromatin remodeler and its relationship to developmental gene expression in Dictyostelium. Genome Res.

[18] Bai L, Morozov AV (2010). Gene regulation by nucleosome positioning. Trends Genet.

[19] Lieberman-Aiden E (2009). Comprehensive mapping of long-range interactions reveals folding principles of the human genome. Science.

[20] Lupianez DG (2015). Disruptions of topological chromatin domains cause pathogenic rewiring of gene-enhancer interactions. Cell.

[21] Rodriguez J (2019). Intrinsic dynamics of a human gene reveal the basis of expression heterogeneity. Cell.

[22] Behera V (2018). Exploiting genetic variation to uncover rules of transcription factor binding and chromatin accessibility. Nat Commun.

[23] Oh E (2017). Transcriptional heterogeneity in the lactase gene within cell-type is linked to the epigenome. Sci Rep.

[24] Jin WF (2015). Genome-wide detection of DNase I hypersensitive sites in single cells and FFPE tissue samples. Nature.

[25] Ohlsson R, Renkawitz R, Lobanenkov V (2001). CTCF is a uniquely versatile transcription regulator linked to epigenetics and disease. Trends Genet.

[26] Fedoriw AM, Stein P, Svoboda P, Schultz RM, Bartolomei MS (2004). Transgenic RNAi reveals essential function for CTCF in H19 gene imprinting. Science.

[27] Wan LB (2008). Maternal depletion of CTCF reveals multiple functions during oocyte and preimplantation embryo development. Development.

[28] Heath H (2008). CTCF regulates cell cycle progression of alphabeta T cells in the thymus. EMBO J.

[29] Filippova GN (1996). An exceptionally conserved transcriptional repressor, CTCF, employs different combinations of zinc fingers to bind diverged promoter sequences of avian and mammalian c-myc oncogenes. Mol Cell Biol.

[30] Klenova EM (1993). Ctcf, a conserved nuclear factor required for optimal transcriptional activity of the chicken C-Myc gene, is an 11-Zn-finger protein differentially expressed in multiple forms. Mol Cell Biol.

[31] Vostrov AA, Quitschke WW (1997). The zinc finger protein CTCF binds to the APBbeta domain of the amyloid beta-protein precursor promoter. Evidence for a role in transcriptional activation. J Biol Chem.

[32] Bell AC, West AG, Felsenfeld G (1999). The protein CTCF is required for the enhancer blocking activity of vertebrate insulators. Cell.

[33] Saitoh N (2000). Structural and functional conservation at the boundaries of the chicken beta-globin domain. EMBO J.

[34] Hark AT (2000). CTCF mediates methylation-sensitive enhancer-blocking activity at the H19/Igf2 locus. Nature.

[35] Phillips JE, Corces VGCTCF (2009). Master weaver of the genome. Cell.

[36] Cuddapah S (2009). Global analysis of the insulator binding protein CTCF in chromatin barrier regions reveals demarcation of active and repressive domains. Genome Res.

[37] Dekker J, Mirny L (2016). The 3D genome as moderator of chromosomal communication. Cell.

[38] Sexton T (2012). Three-dimensional folding and functional organization principles of the Drosophila genome. Cell.

[39] Phillips-Cremins JE (2013). Architectural protein subclasses shape 3D organization of genomes during lineage commitment. Cell.

[40] Wang M (2018). Putative bovine topological association domains and CTCF binding motifs can reduce the search space for causative regulatory variants of complex traits. BMC Genomics.

[41] Aitken SJ (2018). CTCF maintains regulatory homeostasis of cancer pathways. Genome Biol.

[42] Nora EP (2017). Targeted degradation of CTCF decouples local insulation of chromosome domains from genomic compartmentalization. Cell.

[43] Tiana G (2016). Structural fluctuations of the chromatin fiber within topologically associating domains. Biophys J.

[44] Dowen JM (2014). Control of cell identity genes occurs in insulated neighborhoods in mammalian chromosomes. Cell.

[45] Narendra V (2015). CTCF establishes discrete functional chromatin domains at the Hox clusters during differentiation. Science.

[46] Fudenberg G (2016). Formation of chromosomal domains by loop extrusion. Cell Rep.

[47] Rao SSP (2017). Cohesin loss eliminates all loop domains. Cell.

[48] Nichols MH, Corces VG (2015). A CTCF code for 3D genome architecture. Cell.

[49] de Wit E (2015). CTCF binding polarity determines chromatin looping. Mol Cell.

[50] Guo Y (2015). CRISPR inversion of CTCF sites alters genome topology and enhancer/promoter function. Cell.

[51] Zuin J (2014). Cohesin and CTCF differentially affect chromatin architecture and gene expression in human cells. Proc Natl Acad Sci USA.

[52] Barski A (2007). High-resolution profiling of histone methylations in the human genome. Cell.

[53] Monahan K (2012). Role of CCCTC binding factor (CTCF) and cohesin in the generation of single-cell diversity of protocadherin-alpha gene expression. Proc Natl Acad Sci USA.

[54] Sanyal A, Lajoie BR, Jain G, Dekker J (2012). The long-range interaction landscape of gene promoters. Nature.

[55] Merkenschlager M, Nora EP (2016). CTCF and cohesin in genome folding and transcriptional gene regulation. Annu Rev Genomics Hum Genet.

[56] Busslinger GA (2017). Cohesin is positioned in mammalian genomes by transcription, CTCF and Wapl. Nature.

[57] Liu Z, Scannell DR, Eisen MB, Tjian R (2011). Control of embryonic stem cell lineage commitment by core promoter factor, TAF3. Cell.

[58] Zhao HL (2015). PARP1- and CTCF-mediated interactions between active and repressed chromatin at the lamina promote oscillating transcription. Mol Cell.

[59] Bell AC, Felsenfeld G (2000). Methylation of a CTCF-dependent boundary controls imprinted expression of the Igf2 gene. Nature.

[60] Wang H (2012). Widespread plasticity in CTCF occupancy linked to DNA methylation. Genome Res.

[61] Liu XS (2016). Editing DNA methylation in the mammalian genome. Cell.

[62] Tremblay KD, Saam JR, Ingram RS, Tilghman SM, Bartolomei MS (1995). A paternal-specific methylation imprint marks the alleles of the mouse H19 gene. Nat Genet.

[63] Reik W (1995). Imprinting mutations in the Beckwith–Wiedemann syndrome suggested by an altered imprinting pattern in the Igf2-H19 domain. Hum Mol Genet.

[64] Stadler MB (2012). DNA-binding factors shape the mouse methylome at distal regulatory regions (vol 480, pg 490, 2011). Nature.

[65] Matsuzaki H, Okamura E, Fukamizu A, Tanimoto K (2010). CTCF binding is not the epigenetic mark that establishes post-fertilization methylation imprinting in the transgenic H19 ICR. Hum Mol Genet.

[66] Flavahan WA (2016). Insulator dysfunction and oncogene activation in IDH mutant gliomas. Nature.

[67] Handoko L (2011). CTCF-mediated functional chromatin interactome in pluripotent cells (vol 43, pg 630, 2011). Nat Genet.

[68] Katainen R (2015). CTCF/cohesin-binding sites are frequently mutated in cancer. Nat Genet.

[69] Hnisz D (2016). Activation of proto-oncogenes by disruption of chromosome neighborhoods. Science.

[70] Lam FH, Steger DJ, O’Shea EK (2008). Chromatin decouples promoter threshold from dynamic range. Nature.

[71] Schones DE (2008). Dynamic regulation of nucleosome positioning in the human genome. Cell.

[72] Shivaswamy S (2008). Dynamic remodeling of individual nucleosomes across a eukaryotic genome in response to transcriptional perturbation. PLoS Biol.

[73] Hu GQ (2011). Regulation of nucleosome landscape and transcription factor targeting at tissue-specific enhancers by BRG1. Genome Res.

[74] Ostuni R (2013). Latent enhancers activated by stimulation in differentiated cells. Cell.

[75] Fu YT, Sinha M, Peterson CL, Weng ZP (2008). The insulator binding protein CTCF positions 20 nucleosomes around its binding sites across the human genome. PLoS Genet.

[76] Sherwood RI (2014). Discovery of directional and nondirectional pioneer transcription factors by modeling DNase profile magnitude and shape. Nat Biotechnol.

[77] Pan Q, Shai O, Lee LJ, Frey J, Blencowe BJ (2008). Deep surveying of alternative splicing complexity in the human transcriptome by high-throughput sequencing. Nat Genet.

[78] de la Mata M (2003). A slow RNA polymerase II affects alternative splicing in vivo. Mol Cell.

[79] Meissner A (2008). Genome-scale DNA methylation maps of pluripotent and differentiated cells. Nature.

[80] Maunakea AK, Chepelev I, Cui KR, Zhao KJ (2013). Intragenic DNA methylation modulates alternative splicing by recruiting MeCP2 to promote exon recognition. Cell Res.

[81] Wada Y (2009). A wave of nascent transcription on activated human genes. Proc Natl Acad Sci USA.

[82] Paredes SH, Melgar MF, Sethupathy P (2013). Promoter-proximal CCCTC-factor binding is associated with an increase in the transcriptional pausing index. Bioinformatics.

[83] Stadhouders R (2012). Dynamic long-range chromatin interactions control Myb proto-oncogene transcription during erythroid development. EMBO J.

[84] Shukla S (2011). CTCF-promoted RNA polymerase II pausing links DNA methylation to splicing. Nature.

[85] Gibcus JH (2018). A pathway for mitotic chromosome formation. Science.

[86] Nagano T (2017). Cell-cycle dynamics of chromosomal organization at single-cell resolution. Nature.

[87] Earnshaw WC, Laemmli UK (1983). Architecture of metaphase chromosomes and chromosome scaffolds. J Cell Biol.

[88] Naumova N (2013). Organization of the mitotic chromosome. Science.

[89] Oomen ME, Hansen AS, Liu Y, Darzacq X, Dekker J (2019). CTCF sites display cell cycle-dependent dynamics in factor binding and nucleosome positioning. Genome Res.

[90] Cattoglio C (2019). Determining cellular CTCF and cohesin abundances to constrain 3D genome models. Elife.

[91] Cai Y (2018). Experimental and computational framework for a dynamic protein atlas of human cell division. Nature.

[92] Sekiya T, Murano K, Kato K, Kawaguchi A, Nagata K (2017). Mitotic phosphorylation of CCCTC-binding factor (CTCF) reduces its DNA binding activity. FEBS Open Bio.

[93] Dephoure N (2008). A quantitative atlas of mitotic phosphorylation. Proc Natl Acad Sci USA.

[94] Dovat S (2002). A common mechanism for mitotic inactivation of C2H2 zinc finger DNA-binding domains. Gene Dev.

[95] Jantz D, Berg JM (2004). Reduction in DNA-binding affinity of Cys2His2 zinc finger proteins by linker phosphorylation. Proc Natl Acad Sci USA.

[96] Altschuler SJ, Wu LF (2010). Cellular heterogeneity: do differences make a difference?. Cell.

